# A Drug-Eluting Injectable NanoGel for Localized Delivery of Anticancer Drugs to Solid Tumors

**DOI:** 10.3390/pharmaceutics15092255

**Published:** 2023-08-31

**Authors:** Brent Godau, Sadaf Samimi, Amir Seyfoori, Ehsan Samiei, Tahereh Khani, Parvaneh Naserzadeh, Alireza Hassani Najafabadi, Emal Lesha, Keivan Majidzadeh-A, Behnaz Ashtari, Gabriel Charest, Christophe Morin, David Fortin, Mohsen Akbari

**Affiliations:** 1 Laboratory for Innovations in MicroEngineering (LiME), Department of Mechanical Engineering, University of Victoria, Victoria, BC V8P 5C2, Canada; 2 Center for Advanced Materials and Related Technology (CAMTEC), University of Victoria, Victoria, BC V8P 5C2, Canada; 3 Preclinical Lab., Core Facility, Tehran University of Medical Sciences, Tehran 1417755354, Iran; 4 Endocrine Research Center, Institute of Endocrinology and Metabolism, Iran University of Medical Sciences, Tehran 88945173, Iran; 5 Terasaki Institute for Biomedical Innovations, Los Angeles, CA 90050, USA; hassania@terasaki.org; 6 Department of Neurosurgery, University of Tennessee Health Science Center, Memphis, TN 38163, USA; 7 Genetics Department, Breast Cancer Research Center, Motamed Cancer Institute, ACECR, No. 146, South Gandhi Ave., Vanak Sq., P.O. BOX 1517964311, Tehran 1684613114, Iran; 8 Department of Medical Nanotechnology, Faculty of Advance Technologies in Medicine, Iran University of Medical Sciences, Tehran 1449614535, Iran; 9 Department of Surgery, Faculty of Medicine, Université de Sherbrooke, Sherbrooke, QC J1K 2R1, Canadasina81@gmail.com (C.M.); david.fortin@usherbrooke.ca (D.F.)

**Keywords:** localized therapy, shear-thinning hydrogel, cancer therapy, glioblastoma, breast cancer

## Abstract

Systemically administered chemotherapy reduces the efficiency of the anticancer agent at the target tumor tissue and results in distributed drug to non-target organs, inducing negative side effects commonly associated with chemotherapy and necessitating repeated administration. Injectable hydrogels present themselves as a potential platform for non-invasive local delivery vehicles that can serve as a slow-releasing drug depot that fills tumor vasculature, tissue, or resection cavities. Herein, we have systematically formulated and tested an injectable shear-thinning hydrogel (STH) with a highly manipulable release profile for delivering doxorubicin, a common chemotherapeutic. By detailed characterization of the STH physical properties and degradation and release dynamics, we selected top candidates for testing in cancer models of increasing biomimicry. Two-dimensional cell culture, tumor-on-a-chip, and small animal models were used to demonstrate the high anticancer potential and reduced systemic toxicity of the STH that exhibits long-term (up to 80 days) doxorubicin release profiles for treatment of breast cancer and glioblastoma. The drug-loaded STH injected into tumor tissue was shown to increase overall survival in breast tumor- and glioblastoma-bearing animal models by 50% for 22 days and 25% for 52 days, respectively, showing high potential for localized, less frequent treatment of oncologic disease with reduced dosage requirements.

## 1. Introduction

Treating cancer using chemotherapeutic agents has been a major clinical challenge. Significant progress has been made over the past decades to find new targets and formulate novel drugs for cancer therapy; however, treatment success is often hindered by limitations in tissue target access and the need for high doses of the drug to achieve therapeutic efficacy. The main challenge of delivering drugs to target tissues is the ability to account for proper tissue distribution, cellular uptake, and metabolism while maintaining therapeutic efficacy and minimizing systemic toxicity [1].

Localized therapy is an attractive method for delivering chemotherapies directly to the tumor site [2,3]. This approach minimizes off-target toxicities associated with the systemic circulation of chemotherapies while improving drug distribution and bioavailability at the disease site. Moreover, localized therapy overcomes biological barriers, including the blood–brain barrier, and increases the therapeutic options [4]. To this end, numerous implantable drug delivery systems have been developed that act as drug depots for the controlled release of therapeutic agents at the disease site [5,6]. These systems utilize polymeric drug-eluting wafers (e.g., Gliadel wafers) for passive drug delivery or convection-enhanced platforms that infuse drugs into the disease site using a peripheral or implantable pump (e.g., Azlet Osmotic pump) [7]. However, deploying these systems requires expensive surgical procedures, increases patient discomfort, prolongs hospital stays, and increases the chances of complications, such as post-surgical infections. Additionally, repeated therapy using these approaches requires subsequent surgeries or is associated with the risk of backflow, bubble formation, and catheter-associated infections.

Localized delivery of therapeutic payloads using minimally invasive procedures is an attractive alternative that is less expensive and causes less pain and trauma for patients [8]. In this approach, drugs are loaded in injectable delivery vehicles and are delivered to the disease site using medical catheters or hypodermic needles. Polymeric micro- and nanoparticles have been extensively used as injectable drug carriers for delivery of chemotherapies to tumors [9,10]. These carriers serve as drug depots that enable prolonged release profiles while improving drug bioactivity by protecting it from the physiological environment. However, particle dislocation and non-uniform drug distribution post implantation remain significant challenges, limiting their widespread use in the clinic.

Injectable hydrogels have gained interest in recent years as a promising method of localized drug delivery [11,12]. Biodegradable hydrogels that can form gels in situ have been widely utilized for biomedical applications [13]. Notably, shear-thinning hydrogels that experience a decrease in viscosity upon application of shear have shown great promise for delivering a therapeutic payload to the tumor site [14]. Such behavior reduces the injection forces required to pass the gel through narrow catheters or needles. Additionally, shear-thinning hydrogels can readily fill and take the shape of a cavity following tumor resection, providing a suitable interface between the gel and cancerous tissue [15]. The porosity and rheological properties of these gels can be adjusted to control the release kinetics of drug delivery. Recently, shear-thinning hydrogels composed of silicate nanoparticles and hydrogels have been developed for various biomedical applications [11,12,16,17]. The shear-thinning properties of these nanocomposite gels rely on the edge-rim electrostatic interaction between the gel and nano silicate particles. Laponite—two-dimensional (2D) nanoplatelets made of lithium, magnesium, and sodium silicate (~1 nm thickness and 20–50 nm diameter)—has been used in combination with gelatin, alginate, polyethylene glycol (PEG), and silk fibroin for tissue engineering, additive manufacturing, or as tissue adhesives [17,18,19]. Laponite’s electrostatic properties can also act as an effective means for adsorbing doxorubicin (Dox), a potent anticancer drug, enabling a dual-function shear-thinning hydrogel with beneficial properties for catheter injection and electrostatic interactions with Dox that promotes sustained drug release [20].

**Figure 1:**
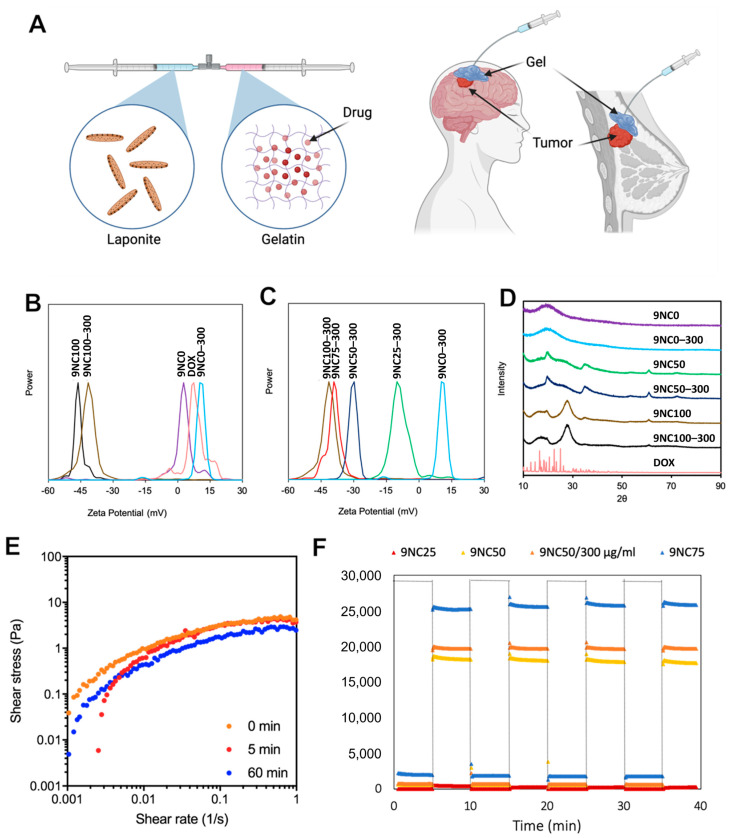
Characterization of drug-loaded nanocomposite hydrogel with shear-thinning and self-healing properties. (**A**) Schematic representation of the drug-loaded nanocomposite formulation. (**B**) Zeta potential analysis reveals strong electrostatic interaction between Laponite and doxorubicin, while weak interaction is observed between gelatin and doxorubicin. (**C**) Variation in the gelatin and Laponite ratio in drug-loaded gels allows control over the gel’s overall charge. (**D**) XRD analysis shows amorphous loading of doxorubicin into the nanocomposite hydrogels. (**E**) Time-dependent shear stress vs. shear rate plots demonstrate the shear-thinning behavior of the nanocomposite hydrogel at 0 min, 5 min, and 60 min after loading. (**F**) Self-healing performance is evident as the nanocomposite hydrogel shows full recovery after repeated exposure to high and low strain.

In this study, we report a facile approach to using Laponite/gelatin shear-thinning hydrogel (STH) for chemotherapy delivery to cancer cells (Figure 1A). Previously, we showed that the nanocomposite hydrogel used herein exhibited excellent biocompatibility and injectability profiles in vitro and in rodent and porcine models [12]. Here, we expanded the potential applications of this material by showcasing the feasibility and advantages of using the STH as an effective chemotherapy platform for controlled drug delivery of Dox. We evaluated the effect of Dox on the rheological, swelling, and degradation properties of STH and assessed the anticancer efficacy and biocompatibility of this material in vitro and in vivo.

## 2. Methods

### 2.1. Formulation & Naming System

In this study, gelatin and Laponite solutions were combined to achieve a final concentration (wt%) with specific gelatin:Laponite ratios. The naming convention for the formulations follows the pattern XNCY, where X represents the final combined concentration, Y represents the percentage of Laponite solution in the nanocomposite (NC), and NC refers to nanocomposite [21]. For instance, a formulation composed of 25 parts 9% Laponite solution and 75 parts 9% gelatin solution is designated as 9NC25. To load doxorubicin (Dox) into the shear-thinning hydrogel (STH), a higher concentration solution of gelatin is diluted to the desired final concentration using a Dox solution. The samples containing Dox are named based on the amount of loaded drug per gram of STH, denoted as mass (µg) of Dox per gram of STH. For example, the STH with 9NC25 composition loaded with 150 µg of Dox per gram of STH is referred to as 9NC25-150.

### 2.2. STH Formulation

An 18% (*w*/*v*) stock solution of type A gelatin from porcine skin (Sigma Aldrich, 300 Bloom, St. Louis, MI, USA) was prepared by dissolving the gelatin in Milli-Q ultrapure water at 40 °C and subsequently vortexing to achieve a homogenous solution. Laponite XLG (BYK Performance Additives, Wesel, Germany) was used to create a 6% or 9% (*w*/*v*) solution, and it was mixed in pre-cooled Milli-Q ultrapure water at 4 °C to delay gelation and ensure homogeneity. The gelatin and Laponite solutions were combined in their respective ratios, with the gelatin being diluted by a factor of 2 using Milli-Q ultrapure water, and the mixture was vortexed to create various compositions of the shear-thinning hydrogel (STH). For STHs containing doxorubicin (Dox) (Sigma Aldrich, St. Louis, MI, USA), a solution of Dox HCl was used to dilute the gelatin solution during the gel mixing process. The gels were then stored at 37 °C for a minimum of 48 h to allow for Dox intercalation within the hydrogel.

### 2.3. Zeta Potential

The zeta potential of each sample was determined at room temperature using a Brookehaven BI-ZR3 Zeta Potential Analyzer (Brookehaven Instruments, Holtsville, NY, USA) equipped with a 660 nm wavelength laser. The shear-thinning hydrogel (STH) samples were prepared following the previously mentioned method but with a reduced concentration of 0.2% to achieve a less viscous solution.

### 2.4. X-ray Diffraction

As previously described, nanocomposites were prepared, and the final concentration of doxorubicin (Dox) in the Dox-loaded nanocomposites was set to 300 µg/g of gel. Subsequently, all samples underwent freeze-drying by storing them in a −80 °C freezer for 24 h, followed by lyophilization for 2 days. The resulting samples were ground into a powder and subjected to analysis using a PANalytical Empyrean X-ray Diffractometer (Spectris, London, UK) at room temperature, operating with a current of 40 mA and a voltage of 45 kV, over a diffraction angle range of 10–90° (2θ).

### 2.5. Scanning Electron Microscopy

Nanocomposites with diverse combinations of gelatin and Laponite, along with varying concentrations of doxorubicin (Dox), were prepared following the previously mentioned procedure. Subsequently, freeze-drying was employed for sample preparation, as described earlier. The imaging of the samples was carried out using a Hitachi S-4800 Field Emission Scanning Electron Microscope (FESEM) (Hitachi, Tokyo, Japan). Pore size was measured using ImageJ software (version 1.51).

### 2.6. In Vitro Release Study in Well plates

STH with various compositions were prepared in 0.25 g aliquots, each containing 150 µg/g Dox, and submerged in 1 mL of 0.1 M phosphate-buffered saline (PBS) with a pH of 7.4. All samples were kept at 37 °C and gently agitated by vortexing while removing the supernatant. Prior to supernatant removal, the samples were centrifuged on a mini centrifuge at 2000 G for 5 min. The release of Dox from the STH was quantified by extracting the entire 1 mL of supernatant and subjecting it to fluorescence spectroscopy. A 480 nm wavelength excitation and a 598 nm emission were utilized for analysis.

### 2.7. In Vitro Drug Toxicity Studies in 2D and 3D Cultures

U87 Human glioblastoma cells (ATCC: HTB-14) and MCF7 breast adenocarcinoma cells (ATCC: HTB-22) were cultivated in 25 cm^2^ flasks using high glucose Dulbecco’s Modified Eagle Medium (DMEM) supplemented with 10% fetal bovine serum (FBS) and 1% penicillin/streptomycin. For U87 cells, 4 µg/mL puromycin was incorporated into the medium. The cell cultures were maintained at 37 °C with 95% relative humidity and 5% CO_2_. A medium change was performed every other day throughout the culture period.

### 2.8. Free Dox Cell Viability

Cells were seeded at a density of 10,000 cells per well into 96-well plates (VWR, Radnor, PA, USA) and cultured in 0.2 mL of culture medium for 24 h. Following this, the medium was replaced, and different concentrations of Dox (0.1, 1, 10, and 100 µg/mL) were added to the culture medium. The plates were maintained under the same conditions as mentioned earlier for cell culture, and the cell viability of each condition was assessed at 24 h, 48 h, 72 h, and 96 h using a Presto Blue assay.

### 2.9. Dox-Loaded STH-Treated Cell Viability

Cells were seeded into 24-well plates (Sarstedt, Numbrecht, Germany) at a density of 25,000 cells per well and cultured with 1 mL of medium for 24 h. Subsequently, the cells were exposed to 0.1 g samples of doxorubicin (Dox)-loaded STHs placed in a 12 mm, 12 µm pore size cell-culture insert (Millipore Sigma, 12 mm diameter, 12 µm pore size, Burlington, MA, USA). The well medium was replaced with 0.6 mL of fresh medium, while the insert received 0.4 mL of fresh medium containing the Dox-loaded STH. The plates were then stored under the same conditions as mentioned earlier for cell culture. The cell viability of each condition was evaluated at 24 h, 48 h, 72 h, and 96 h using a Presto Blue assay.

### 2.10. Three-Dimensional (3D) Microfluidic Culture

The 3D microfluidic devices were fabricated following our group’s previously described method [22]. To summarize, polydimethylsiloxane (PDMS) was used to create the microfluidic device, which was then bonded to 25 mm coverslips. The microchannel architecture of the tumor compartment and delivery side channels was formed by casting PDMS on an SU-8 master mold, followed by baking at 80 °C for 2 h. Biopsy punches were utilized to create 5 mm medium wells and 1 mm inlets for injecting hydrogel into the tumor compartment. The PDMS piece and the coverslip were plasma treated for 60 s and then bonded, followed by baking at 80 °C for 30 min.

To ensure device sterility and remove potential PDMS debris, the devices were rinsed twice with 70% ethanol and once with 100% ethanol. Subsequently, they were baked at 80 °C for 4 h in a sterile container to completely remove the ethanol. To enhance hydrogel attachment to the microfluidic device, the surface of the tumor compartment was coated with poly-D-lysine (PDL). A 1 mg/mL PDL solution in DI water was pipetted into the tumor compartment and incubated in a humid incubator at 37 °C for 1 h. After the incubation, the PDL solution was removed, and the microchannels were rinsed three times with DI water. Finally, the devices were baked at 80 °C for 4 h to restore PDMS hydrophobicity.

### 2.11. Cell Encapsulation in Collagen

U87 cells were cultured on a culture plate until they reached 80% confluency. Following this, the cells were trypsinized, centrifuged, and resuspended in fresh culture medium at 4 °C. Both the hydrogel and cell suspension preparations were performed at 4 °C. An acidic type I collagen solution with a concentration of 10 mg/mL was mixed with NaOH and 10× PBS to create a buffered 8 mg/mL collagen solution.

The cell suspension was combined with the collagen solution, and the volume was adjusted using culture medium to achieve a final solution with a collagen concentration of 4 mg/mL and a cell density of 1 million cells/mL. Subsequently, the cell/collagen solution was pipetted into the tumor compartment of the microfluidic device and incubated in a humid incubator at 37 °C for 45–60 min until the collagen completely gelled. Culture medium was then added to the wells, and the model was incubated overnight in preparation for the subsequent experimentation.

### 2.12. STH Injection in a Microfluidic Device

Injection of STH into the delivery side channel of the glioblastoma-on-chip model was performed using a syringe pump at a constant flow rate to ensure consistency in the delivery between different devices. STH was loaded in a 3 mL syringe, and the outlet was connected to a 0.8 mm diameter Tygon tube. The flow rate in the syringe pump was set to 100 µL/min, and pumping was started until a steady flow of STH was observed. The medium from one of the 2 delivery channels was removed, and the tube outlet was held at the inlet of the microchannel. The flow of STH was continued until the microchannel was uniformly filled and the two side wells were partially loaded with STH. The remaining space in the wells was filled with culture medium to maintain the STH hydrated throughout the experiment.

### 2.13. Diffusion Test with the Microfluidic Device

To investigate the diffusion of Dox into the tumor compartment of the glioblastoma-on-chip model, two approaches were employed: using free Dox dissolved in DPBS and Dox-loaded shear-thinning hydrogel (Dox-STH). The model was initially prepared with cell-free collagen and kept hydrated with DPBS. For the free drug experiment, a 150 mg/mL Dox solution in DPBS was prepared, and the DPBS from one of the delivery channels in the model was replaced with the Dox solution. Real-time imaging using the red channel of a Zeiss microscope was promptly initiated. Images were captured every 60 s for a total of 100 min, with an exposure time of 20 ms. The fluorescence intensity was measured along an axis covering a section of the delivery channel and the entire width of the tumor compartment channel. The measured fluorescence intensity was then normalized using the average value in the delivery channel. In the case of Dox-STH, the DPBS in the delivery channel was removed, and the STH was injected following the procedure mentioned earlier. Imaging using the red channel was conducted every 4 h for a total duration of 4 days. Fluorescence intensity measurements were taken across the width of the tumor compartment and a section of the delivery channel, which were later normalized using the average intensity in the delivery channel.

### 2.14. COMSOL Brain-Tumor-on-a-Chip Dox Diffusion Simulation

COMSOL Multiphysics was employed to simulate the drug diffusion profile to the gel network inside a microfluidic device. Drug diffusion was investigated for both free drug and drug-loaded gel to study the time-dependent transport of the drug in a porous medium. The model was designed by establishing three 2D network planes of a microfluidic device. The microfluidic device consists of three channels, and the model was established by simulating the middle and side channels with hydrogel and drug solution for varying conditions. The simulation of the free drug diffusion was performed for 0, 40, and 100 min after inserting the solution into the side channel and releasing it into the middle channel with a 2000 μm length. In addition, the drug-loaded STH model was simulated for 96 h after injection of the drug-loaded hydrogel into the side channel and releasing the drug into the middle channel. The concentration for both of these simulations was 0.0337 mol/L.

### 2.15. In Vivo Antitumor Activity of Dox-Loaded STHs

We evaluated the safety and performance of STH in two animal models of orthotopic breast and brain tumors.

#### 2.15.1. Rat Glioblastoma Model and STH Implantation

For all procedures, male Fischer rats (Charles River Laboratories, Saint-Constant, QC, Canada) were anesthetized with Isoflurane 3%, 2 L/min. The experimental protocol (2018-2142, 447-18) was approved by the institutional ethical committee and conformed to the Canadian Council on Animal Care regulations. For the implantation procedure, confluent F98 glial cells were suspended in non-supplemented warm MEM at a concentration of 2000 cells/µL. The implantation (10,000 cells in 5 µL) was performed as described by Mathieu et al. [23]. In total, 31 rats divided into the following 5 groups were used to evaluate the efficiency of three STH formulations compared to intravenous (IV) doxorubicin treatment: Doxorobicin IV (*n* = 6), Control “Sham” (pooled STH 3NC50 + 6NC50 + 9NC50 without doxorubicin) (*n* = 9), STH 3NC50 + Dox (*n* = 6), STH 6NC50 + Dox (*n* = 6) and STH 9NC50 + Dox (*n* = 4).

Ten days after tumor cell implantation, 15 µL of Dox-loaded STH was injected through the same burr hole used for tumor implantation. The STH was injected along the needle track in 5 min from 5.5 mm deep, retracting 1 mm each minute. The burr hole was then closed with bone wax, the scalp was sutured, and anesthetic cream was spread on the suture. Animals were kept with food and water ad libitum until the endpoint was reached.

Animal monitoring, including weight measurement, mobility, coordination, loss of self-grooming (periocular secretion accumulation), and landing ability, was performed daily. In agreement with the ethical committee regulations, the experimental endpoint for survival was established when the animals lost a maximum of 20% of their initial weight or when one of the monitored functions reached a score of 4/10. At this point, animals were anesthetized, and 4% paraformaldehyde (PFA) was infused by intra-cardiac route to fix the brain tissue. The brain was removed by craniotomy to corroborate the presence of a tumor.

#### 2.15.2. Mice Breast Tumor Model and STH Implantation

We used Balb/c female mice (Pasteur Institute, Iran) weighing 18 g at the age of 8–10 weeks. Each mouse was anesthetized by an injection of xylazine (100 mg/kg) and ketamine (15 mg/kg) followed by subcutaneous injection in the right flank area with 3 × 10^7^ 4T1 mice breast cancer cell line suspended in 0.1 mL of normal saline. The tumor volume in both models was calculated as (length × width^2^)/2, and the change in the tumor volume was monitored as a function of time.

Tumor-bearing mice were randomly divided into 4 groups (*n* = 5), including (1) mice treated with PBS (5 mL/kg) as a negative control (without treatment), (2) mice treated with 300 Dox-loaded STH, (3) mice that received a blank STH hydrogel (2 mL/kg); and (4) mice injected with a Dox solution at a dosing amount of 50 mg/kg. All experimental interventions started after 10 days of injection, and each group’s tumor size reached an approximate 150 mm^3^. The experimental intervention consisted of subcutaneous injections of drug-encapsulated hydrogel or blank hydrogel at four points around the tumor. Free Dox was administered intraperitoneally into the 4T1 tumor-bearing mice every 2 days up to 4 days after the start of the intervention. In vivo antitumor behavior of the Dox-encapsulated hydrogel compared with the blank gel and free Dox group was evaluated by measuring the tumor size in each animal, recorded every 3 days following initial treatment. The body weight of cancerous mice was used to measure each intervention condition. Moreover, the tumor-bearing mice and rats’ survival rate was evaluated up to 60 days post treatment for each experimental group.

#### 2.15.3. In Vivo Fluorescent Imaging

Fluorescent distribution intensity of the Dox around the tumor and the whole body in mice breast cancer models were measured using an in vivo fluorescent imaging system at time intervals of 1, 2, 4, 24, and 48 h post injection and followed by anesthetization of each group of mice.

#### 2.15.4. Histopathological Studies

Four weeks post STH implantation, 6 rats from each group were randomly selected and sacrificed. Tissues from the tumor site and the liver and heart were collected and immersed in 10% formalin for 12 h. Subsequently, the tissues were dehydrated with gradient ethanol and finally embedded in paraffin blocks. Afterward, the paraffin blocks were sectioned at a thickness of 4 mm and stained with hematoxylin/eosin (H&E). The histopathological observations were carried out using an Olympus X51 microscope (Olympus Corporation, Tokyo, Japan).

#### 2.15.5. Mitochondrial Preparation and SDH Assay

For conducting the SDH assay in the brain tumor experimental conditions, following rat scarification, the target tissue was removed and morcellated with scissors and placed in a mannitol solution. The tissue was then homogenized with a mortar and pestle and centrifuged at 1500× *g* for 12 min, and the pellet was discarded. The supernatant was then centrifuged at 12,500× *g* for 10 min. The resulting pellet was washed in the isolation medium (75 mM sucrose, 0.225 M D-mannitol, and 0.2 mM EDTA) with a pH = 7.4 and was centrifuged once more at 12,500× *g* for 10 min. The resulting pellets were suspended in a Tris buffer (containing 0.05 M Tris-HCl, 0.25 M sucrose, 20 mM KCl, 2.0 mM MgCl_2_, and 1.0 mM Na_2_HPO_4_) with a pH of 7.4.

### 2.16. Statistical Analyses

Data exclusion was done with a confidence of 0.001 before doing Kaplan–Meier survival curves, which were analyzed by the Log-Rank test. *p* values under 0.05 were considered statistically significant.

## 3. Results and Discussion

### 3.1. Characterization of Dox-Loaded STH

#### 3.1.1. Zeta Potential

The zeta potential values of Laponite (9NC100), gelatin (9NC0), and Dox hydrochloride were determined to be −46.22, 2.73, and 7.44 mV, respectively, as depicted in Figure 1B. The inclusion of Dox into both gelatin and Laponite led to an observable increase in the zeta potential, which is consistent with its positively charged nature (Figure 1B). The total charge of the system is determined by the ratio of gelatin to Laponite when all three components of the STH are combined. As depicted in Figure 1C, varying Laponite ratios at 25%, 50%, and 75% led to a decrease in the overall charge of the STH loaded with Dox to −10.15, −29.74, and −39.14 mV, correspondingly. The net charges of Dox-loaded STHs and the constituent elements of the system are indicative of the electrostatic interactions that take place within the STH. The presence of Laponite, a negatively charged substance, in the STH enables the manipulation of drug release kinetics by exploiting the attraction between the carrier and the positively charged drug, Dox.

#### 3.1.2. Powder X-ray Diffraction

X-ray diffraction (XRD) analysis was conducted to investigate the loading behavior of Dox into the STH. In its dry form, Dox exhibited crystalline salt characteristics, indicated by distinct sharp peaks in the XRD pattern between 10–30° (Figure 1D). However, both 9NC0 and 9NC100 formulations displayed an amorphous nature, with a single broad peak at 20.5° for 9NC0 and three broad peaks at 18.2°, 28.5°, and 62.5° for 9NC100. The 50/50 composite, 9NC50, containing 9% gelatin and 9% Laponite, demonstrated a gelatin-specific amorphous peak along with two additional broad peaks at 20.3° and 36.3°, likely attributed to an increase in the basal plane spacing of Laponite due to gelatin’s presence [24].

Upon incorporating Dox into 9NC0, 9NC50, and 9NC100, the sharp peaks of Dox between 10–30° vanished, suggesting that Dox intercalated into the STH and its components in an amorphous state, driven by electrostatic interactions [25,26]. This indicates a strong interaction between Dox and the STH components, further supporting the amorphous nature of the drug within the STH.

#### 3.1.3. Viscoelasticity and Recovery Analysis

To examine the effect of Dox on the rheological properties of STH, shear stress vs. shear rate sweeps of drug-loaded 9NC75 were monitored using shear rates in the range of 0.001 to 1 s^−1^. The high shear rate used in this study was chosen to emulate unassisted and manual delivery of the gel during minimally invasive procedures. Further, the temporal dependence of STH injectability was assessed by collecting shear rate curves over one hour to determine whether there were significant changes in the material’s rheological properties. These properties are crucial for the continued delivery of the STH throughout the operation window. Notably, maintaining injectability over time will avoid challenges associated with catheter occlusion encountered with ethylene vinyl alcohol and cyanoacrylate-based embolic agents and provide physicians with sufficient time to ensure accurate delivery and safe removal of the catheter. Shear-thinning behavior was evident in all injection conditions, with slopes decreasing as the shear rate was increased with no significant difference between different conditions (Figure 1E). Further, measurement of dynamic changes in rheological properties of STH revealed an insignificant change in shear rate curves of the material immediately, 5 min, and 60 min after loading, demonstrating that the shear-thinning behavior of the STH was preserved even after extended periods without applied flow. Such property enables more precise material deployment with minimum non-target embolization, as can occur with other liquid embolics and coils.

We conducted mechanical stability characterization of drug-loaded shear-thinning hydrogel (STH) under cyclic oscillatory strain conditions with high (100%) and low (1%) strains. The primary focus was on evaluating the modulus recovery after subjecting the gel to high strains. Rapid recovery of the injectable gel’s solid-like behavior was observed across all samples following exposure to high-strain conditions. This instantaneous recovery is crucial to prevent material fragmentation, which could potentially lead to off-target embolization once the material is delivered through a catheter or needle tip. Interestingly, the incorporation of doxorubicin (Dox) into the STH, especially in 9NC50, showed a slightly increased storage modulus (Figure 1E). This phenomenon could be attributed to electrostatic interactions between Laponite and Dox, as discussed in our zeta potential analysis.

#### 3.1.4. Scanning Electron Microscopy

Scanning electron microscopy (SEM) images of the STH with varying gelatin:Laponite ratios and Dox concentrations were obtained to observe the impacts of these parameters on the microscopic pore size of STHs. The pore size of hydrogels governs drug transport and degradation of hydrogel. As shown in Supplementary Figure S1A, increasing the ratio of Laponite resulted in a near-linear reduction in pore size from 97.76 µm for STHs with 0% Laponite to 21.17 µm for STHs with 75% Laponite. Adding negatively charged Laponite to positively charged gelatin resulted in crosslinking of the gelatin and introducing gaps between the Laponite platelets. The pore size for 9NC100 was immeasurable with *ImageJ*. A similar effect was seen by increasing the concentration of Dox loaded into the STHs, which correlates with gelatin and Dox having a similar zeta potential. A near-linear pore size reduction was observed when the Dox concentration increased from 0 to 300 µg of Dox per gram of STH (Supplementary Figure S1B).

### 3.2. In Vitro Enzymatic Degradation and Swelling Test

In vitro enzymatic degradation and swelling tests were performed to assess the behavior of STHs under physiological conditions. The degradation study was conducted at 37 °C using collagenase, an enzyme that naturally degrades gelatin, to simulate the degradation profile of the STH within the body. Over the 19-day study period, samples with higher gelatin ratios exhibited an increased degradation rate. For instance, Supplementary Figure S1C illustrates that 9NC25, 9NC50, and 9NC75 had 45%, 72%, and 85% remaining after 19 days, respectively.

The incorporation of oppositely charged Laponite nanoparticles in higher proportions led to enhanced electrostatic interactions within the final STH hydrogel composite. Consequently, hydrogels with higher Laponite percentages exhibited reduced swelling, which corresponded with the microstructural expansion observed in SEM analysis. Specifically, the degree of swelling was 1096%, 1461%, and 1876% for 9NC75, 9NC50, and 9NC25, respectively (Supplementary Figure S1D).

### 3.3. In Vitro Release Study

**Figure 2:**
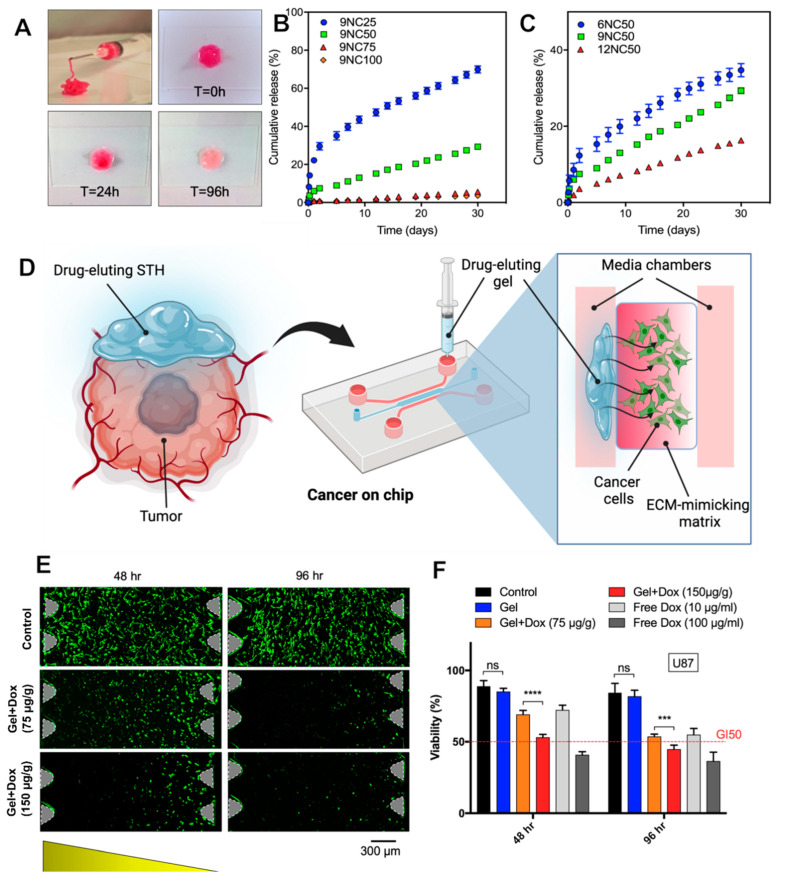
The release profile of the drug is adjusted by changing the nanocomposite formulation. (**A**) Photograph of drug-eluting nanocomposite gel injected from a needle. (**B**) The release rate of the drug decreases as the ratio of nano silicate to overall hydrogel concentration increases. (**C**) The rate of release increases as the overall concentration of the nanocomposite decreases. (**D**) The drug-eluting nanocomposite gel’s anticancer efficiency was evaluated in a 3D microfluidic model of glioblastoma. (**E**) Drug-loaded nanocomposite gel shows dose-dependent antitumor toxicity in the 3D model as evidenced by LIVE staining. (**F**) Anticancer toxicity of the drug-eluting gel was evaluated against various concentrations of the free drug. In all graphs, error bars are the standard deviation of at least three replicates. *** *p* < 0.005 and **** *p* < 0.0001.

Dox was loaded into gelatin by dissolving lyophilized gelatin in deionized water containing 300 µg/mL Dox at 37 °C before mixing with Laponite. To determine release profiles of the drug from STH, drug-loaded gels were immersed in PBS for up to a month. Figure 2A shows time-lapse images of drug-loaded STH over 96 h, demonstrating the depletion of Dox from the gel as evidenced by the change of its color from dark pink to light pink. This nanocomposite maintained its original shape after injection. For effective treatment of tumors, a sustained release of drugs is desired. To this end, the release kinetics of Dox from the STH were investigated with varying ratios of Laponite, overall STH concentrations, and Dox concentrations loaded into the STH (Figure 2B). All release studies were conducted in PBS for 30 days at 37 °C. A high degree of control over the release kinetics was obtained by varying the ratio of Laponite in the STHs. All compositions exhibited a biphasic release with an initial burst release over the first 48 h, followed by a sustained release of the drug at the rate of 0.5% of the loaded drug per day for up to 28 days. The release rates decreased significantly for samples containing higher ratios of Laponite due to the decreased pore size, reduced degradation, and swelling profiles. Another release control parameter is varying overall STH concentration, which decreases the release rate for higher STH concentrations (Figure 2C).

### 3.4. In Vitro Cytotoxicity Studies

#### 3.4.1. MCF7 and U87 Human Glioblastoma Cell Viability

To examine the antiproliferative effect of Dox-loaded STHs on breast and brain tumor cells, MCF7 breast adenocarcinoma and U87 human glioblastoma multiforme cells were cultured and exposed to varying concentrations of free Dox and Dox-loaded 6NC50 and 9NC25. A Presto Blue assay was used to determine cell viability over 96 h. Decreasing MCF7 and U87 cell viability with increasing free Dox concentrations from 0.1 to 100 μg/mL demonstrated dose-dependent viability over four days (Supplementary Figures S2A and S3A). 9NC25 loaded with varying Dox concentrations was used to treat U87 cells. 9NC25 was chosen for its release profile, and 0.1 g samples were weighed to release approximately 0.23, 0.45, and 0.90 µg/mL Dox over 48 h of exposure to 9NC25-75, 9NC25-150, and 9NC25-300, respectively. The second leading candidate for its release profile, 6NC50, was used to treat MCF7 cells and 0.1 g samples were weighed to release approximately 0.15 and 0.50 µg/mL after 48 h of treatment with 6NC50-75 and 6NC50-300, respectively. All three Dox-loaded STHs effectively reduced the viability of U87 and MCF7 cells. However, no statistical difference was seen between the samples containing different Dox concentrations, suggesting minimal variation in dose (Supplementary Figures S2B and 3B). We speculate that this is likely due to the small well plate volume and the requirement of a concentration gradient to release Dox from the STHs via passive diffusion. The ability of Dox-loaded STHs to effectively reduce the viability of U87 and MCF7 cells was also evident in the morphology of the cells. The healthy U87 adhered star-shaped morphology and MCF7 epithelial morphology seen in the control and unloaded STH samples were lost with Dox treatment, showing a rounded morphology for both cell types. As shown in Supplementary Figures S2C and 3C, fluorescent microscopy demonstrates the infiltration of Dox into cells where it is able to force cells into apoptosis. Slightly reduced U87 viability was observed for unloaded STHs compared to the control. However, cell morphology indicated that the cells were healthy. This suggests that the unloaded STH may not kill the cells but may reduce their proliferative capacity. The opposite was true for the MCF7 cells, showing an increased viability over 4 days for the unloaded STH, although the increase was statistically insignificant. The cell viability on day 4 for all Dox-loaded samples was ~5–10%, suggesting that the average concentration of Dox in the medium was close to 1 µg/mL when compared with the free Dox treatment results for both MCF7 and U87. This corresponds with the predictive calculations based on the released data and indicates that the STH has no negative impact on Dox’s ability to induce apoptosis in nearby cancer cells.

#### 3.4.2. In Vitro Brain-Tumor-on-a-Chip Antitumor Activity Study

To bridge the gap between in vitro and in vivo studies, an in vitro brain-tumor-on-a-chip was used to further investigate the antitumor activity of the Dox-loaded STH. The goal was to obtain more accurate data regarding the efficacy of Dox release from the Dox-loaded STH and penetration into dense tumor tissue to kill cancer cells effectively. Three-dimensional tumor tissues were first grown and then treated with free Dox and Dox-loaded STHs, then imaged with live-dead staining to determine cell viability and display cell morphology. As seen in Supplementary Figure S4A, the ability of free Dox to effectively kill U87 cells was significantly reduced when compared to the 2D in vitro study, with the 100 µg/mL concentration reaching 36.5% cell viability after 96 h (compared to ~0% viability in the 2D study after 72 h). This effect is likely due to a reduced ability of Dox to diffuse through collagen and concentrate inside of cells, as would happen faster in the 2D study. A similar effect is seen for treatment with the Dox-loaded 9NC25 (Supplementary Figure S4B). All three samples with varying concentrations of Dox showed a reduced ability to kill U87 cells in the 3D tissue. However, a saturation of Dox was not reached as it was in the 2D study. Increasing the Dox loaded into STHs resulted in a decreased cell viability over 96 h, with final viability of 53.7%, 44.9%, and 34.2% for 9NC25-75, 9NC25-150, and 9NC25-300, respectively. The ability of the Dox-loaded STHs is further exemplified by the rounded morphology of the live and dead cells (Supplementary Figure S4C,D). Moreover, the number of dead cells decreased with increasing distance from the STHs. This is likely due to the slow release of Dox across the entire length of the device, creating a concentration gradient. The unloaded 9NC25-0 sample showed a slightly reduced cell viability compared to the control. However, the cell morphology for the control and the 9NC25-0-treated samples appeared morphologically healthy and spindle-shaped. Moreover, the distribution of dead cells seemed random and not a function of distance from the unloaded STH. An increased cell density is also observed between the 48 and 96 h control and 9NC25-0 samples, demonstrating that the cells are proliferating within the device.

#### 3.4.3. Cancer-on-a-Chip Model

A microfluidic brain-tumor-on-a-chip that was developed in our lab was used for the remainder of the in vitro antitumor activity study. The microfluidic device was designed using SolidWorks and fabricated out of PDMS on a glass slide using soft lithography. As shown in Figure 2D, the device consists of three main channels, each 2000 µm wide, with one side containing reservoirs for cell media and the other two channels having inlets for hydrogel injection. The center channel is injected with U87-laden collagen, and a 3D network of cells is allowed to form. One side channel is reserved for treatment with Dox-loaded STHs, and the other side channel is reserved for cell media (Figure 2D). U87 cells were treated with two concentrations of Dox-loaded STH over 48 and 96 h inside a microfluidic device. As shown in Figure 2E,F, exposing U87 cells to Dox concentrations decreased the cell population after 48 h. Cell population at 75 μg/mL revealed highly dose-dependent cell viability after 48 h.

#### 3.4.4. Computational and Experimental Dox Diffusion Analysis of Brain-Tumor-on-a-Chip

A COMSOL Dox diffusion analysis was employed to ensure that injecting the Dox-loaded STH into a confined volume would not significantly affect the release of Dox from the STH. A collagen diffusion coefficient of 1.8 × 10^−9^ m^2^/s was used in conjunction with the in vitro release data for 9NC25 to predict Dox diffusion across the complete 2000 µm width of the center channel of the microfluidic tumor model. As shown in Supplementary Figure S5, the Dox concentration was predicted to equilibrate 100 min after the injection of free Dox solution into the side channel of the device. This was confirmed by the experimental data. However, the predicted Dox diffusion was much slower for 9NC25, resulting in a slight concentration gradient existing throughout 96 h. This matched with the experimental data, showing a normalized fluorescent intensity ranging from 0.2 to 0.1 across the 2000 µm distance of the center channel.

### 3.5. In Vivo Antitumor Activity of Dox-Loaded STHs

#### 3.5.1. In Vivo Antitumor Activity of Dox-Loaded STHs in Breast Tumor Models

**Figure 3:**
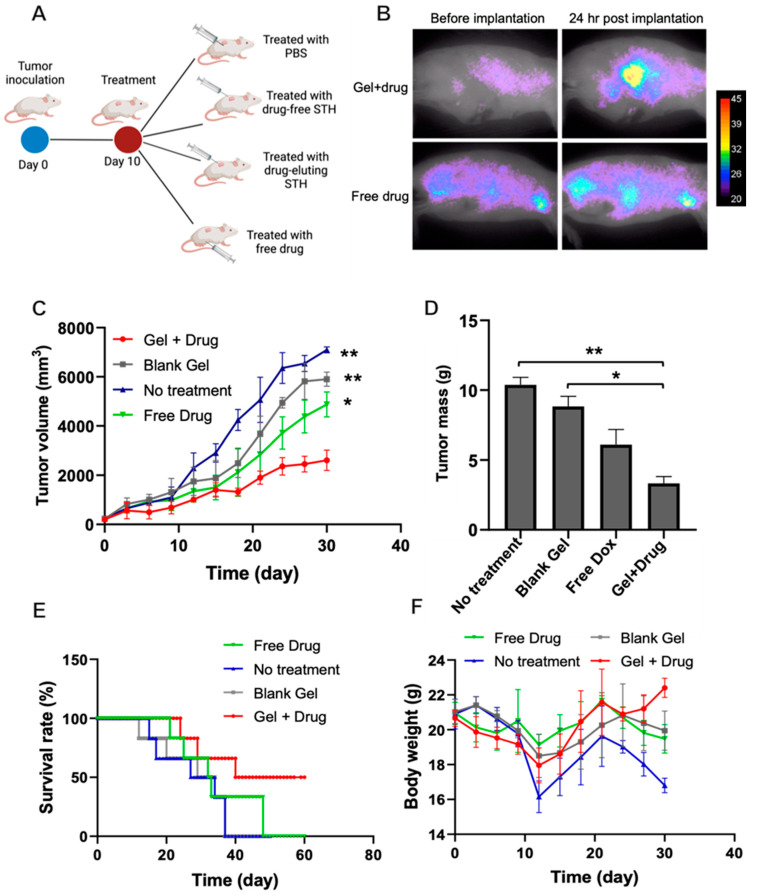
Drug-loaded nanocomposite gel shows antitumor toxicity in a mouse model of breast cancer. (**A**) Animal groups evaluated in this study. (**B**) Localized drug distribution at the tumor site was evaluated using fluorescent imaging. The drug-loaded nanocomposite gel’s antitumor efficacy was assessed by measuring the (**C**) tumor volume over 30 days and (**D**) tumor mass after animals were euthanized on day 30. (**E**) The survival rate of treated animals was evaluated for over 60 days. (**F**) Body weight of the animals for different treatment conditions. In all graphs, error bars are the standard deviation of at least three replicates. * *p* < 0.05 and ** *p* < 0.01.

The treatment effects of our STH gel on breast tumor mice models are depicted in Figure 3. After 30 days of treatment, there was a significant difference in the mean tumor volumes between the treatment and no-treatment groups. Despite evidence of tumor growth in all groups, tumor volume in mice treated with the STH gel was significantly smaller than in the free drug and the blank STH gel groups, demonstrating the efficacy of localized drug release using our injectable hydrogel compared to systemic Dox injection of four times higher concentration. These outcomes are also highlighted with the measurement of tumor mass at the end of the treatment period (Figure 3C). Moreover, mice treated with the STH gel-loaded Dox had a significantly higher survival rate at 60 days and higher body weight despite a lower tumor mass and volume, likely owing to the decreased tumor burden. Moreover, the body weight of the mice treated with systemic Dox decreased sharply after 20 days of treatment, similar to the mice in the no-treatment group. This finding is likely due to the systemic effects of Dox, which has a significant toxicity profile and has been shown to decrease survival in otherwise healthy mice [27].

In vivo Dox biodistribution results in our mice breast tumor model showed localized Dox accumulation at the tumor site, as shown in Figure 3A. Moreover, Dox is gradually released at the tumor site over 24 h, while the systemic Dox injection showed a lack of localized distribution, with diffuse tissue accumulation in other organs shortly after injection. Additionally, over 24 h, the amount of drug in the tissue increased in our STH Dox-loaded injection but decreased significantly in the systemic Dox injection group. This is due to the rapid clearance of the drug with the systemic injection, minimizing the therapeutic effects of the drug and, as often occurs with standard chemotherapy, necessitating multiple drug injections to achieve a therapeutic effect. With localized and controlled drug delivery, as in our current study, slow release of the drug from the gel over time maximizes the amount of drug in the tissue of interest, as such maximizing its therapeutic effect while minimizing systemic drug-related toxicity.

#### 3.5.2. In Vivo Antitumor Activity of Dox-Loaded STHs in Brain Tumor Models

**Figure 4:**
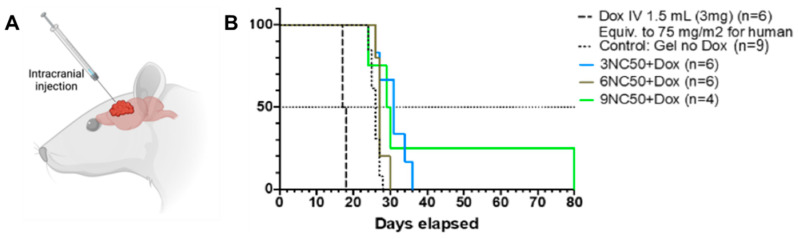
Drug-loaded nanocomposite gel shows antitumor efficiency in an orthotopic rat model of glioblastoma. (**A**) Schematic of intracranial injection of Dox-loaded STH. (**B**) Survival proportion of groups treated with the STH.

The treatment effects of our STH gel on the brain tumor rat models are depicted in Figure 4, with a schematic of the location of gel injection (Figure 4A). The mean survival times in rats treated with blank STH gel were not statistically significant (Log-rank test, *p* = 0.78) for the three formulations (3NC50, 6NC50, and 9NC50), and they were pooled together, as shown in Figure 4. In rats treated with Dox-loaded STH, the fast-release formulation of Dox-loaded 3NC50 and the slow-release formulation of Dox-loaded 9NC50 allowed for a significant survival time extension of 31 days (*p* = 0.0028) and 29.5 days (*p* = 0.017) respectively, compared to the formulations without Dox (26 days). The moderate release formulation of Dox-loaded 6NC50 did not yield a significant extension of survival time (27 days, *p* = 0.06). Neither formulation of Dox-loaded NC50 led to observable systemic toxicity. As observed in the brain tumor rat models, treatment with free Dox to a dose corresponding to the human maximum tolerated dose (MTD of 75 mg/m^2^) translated to a reduced survival time of 17.5 days (Figure 4B), likely due to its high toxicity.

Glioblastoma multiforme consists of over half of primary brain malignancies in adults and has an extremely poor survival rate of 5% in 5 years [28]. Despite advances in surgical techniques and new medical modalities, GBM survival rates have not changed in recent decades. The blood–brain barrier (BBB) has been a therapeutic challenge for many chemotherapeutic agents, including doxorubicin, limiting chemotherapeutic options to mainly Temozolomide (TMZ), the first-line chemotherapeutic agent for GBM. The BBB can be bypassed using local drug delivery, maximizing the effective drug concentration delivered to the tumor while minimizing side effects. Applications of local drug delivery for a brain tumor, especially glioblastoma, have been shown to work with limited success [29]. The Gliadel Wafer, for example, is a commercial product consisting of a biodegradable polymer that releases the drug carmustine and degrades after 6–8 weeks [30]. The wafers, however, are rigid in structure, limiting their application to only resection cavities after surgical resection of the tumor. Local delivery of Dox for treatment of GBM has been previously demonstrated in mouse models with success in tumor growth inhibition [28,29]. In our study, we showed that injection of the STH containing Dox in the tumor via a burr hole site allows for an increase in the mean survival time while minimizing systemic toxicity, allowing for the possibility of using even higher doses of doxorubicin. It is of note that the injected 15 µL of STH-Dox contains only 2.25 µg of Dox, allowing significant tumor control. That is 1200 times less drug than the free Dox IV injection (3 mg). Drug application via STH can thus be beneficial for clinical applications, as doxorubicin can be applied not only via the less invasive procedure, such as burr hole (as compared to craniotomy, thus helping those patients for which tumor resection is not an option), but it can also potentially penetrate better in the resection cavity, reaching penetrative tumor cells that remain despite gross total resection.

#### 3.5.3. Mitochondrial Evaluation of Dox-Loaded STH Cytotoxicity

**Figure 5:**
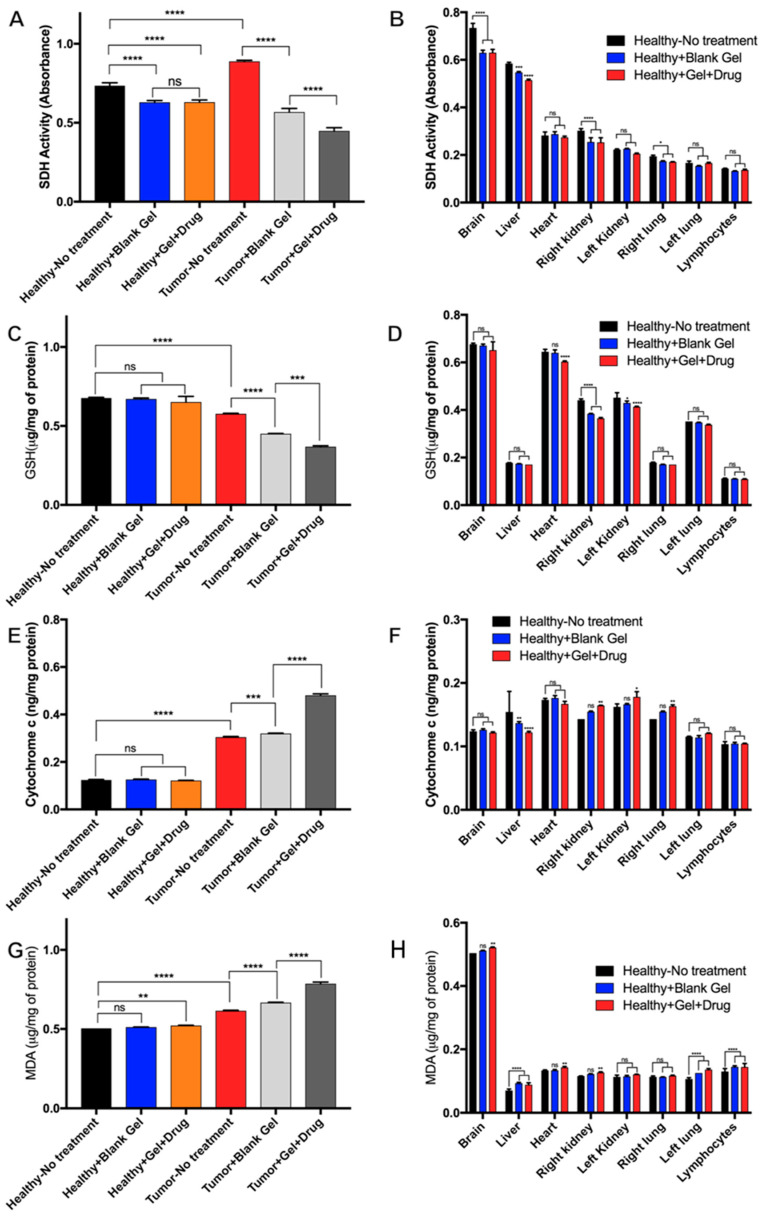
The drug-eluting gel shows minimal local and systemic toxicity using mitochondrial dysfunction analysis in rats. (**A**) Succinate dehydrogenase (SDH) activity in the brain when animals receive different treatments and (**B**) in the liver, heart, kidneys, lungs, and lymphocytes. (**C**) Glutathione activity (GSH) in the brain when animals received different treatments and (**D**) in the liver, heart, kidneys, lungs, and lymphocytes. (**E**) Mitochondrial cytochrome c in the brain when animals received different treatments and (**F**) in the liver, heart, kidneys, lungs, and lymphocytes. (**G**) Malondialdehyde (MDA) in the brain when animals received different treatments and (**H**) in the liver, heart, kidneys, lungs, and lymphocytes. In all graphs, error bars are the standard deviation of at least three replicates. We used one-way ANOVA to compare the results. * *p* < 0.05, ** *p* < 0.01, *** *p* < 0.005, **** *p* < 0.0001.

To assess the cytotoxic effects of the drug-loaded hydrogel, we evaluated mitochondrial succinate dehydrogenase complex II (SDH) levels of blank STH, STH-Dox, and no treatment on the brain tissue (Figure 5A) and the other organs of our experimental rats (Figure 5B). SDH is used as an indicator of cellular activity, with lower levels of SDH activity indicating potential cell death. Figure 5A demonstrates that in the brain tissue, there was a slight decrease in SDH levels. However, it should be noted that there was a similar decrease in SDH activity in both the blank STH and the Dox-loaded STH. Since the decrease was also observed in the blank STH, we hypothesize that it is not Dox related but the result of changes in cellular activity attributed to the hydrogel effect and the possible role of released ions and molecules due to partial degradation of the hydrogel after injection. We evaluated SDH levels in the brain, heart, lungs, kidneys, liver, and lymphocytes to evaluate the cytotoxicity of the Dox-loaded STH in different organs. As illustrated in Figure 5, with the exception of the brain, there is no evidence of a significant decrease in SDH levels in any of the other tissue.

Evaluation of GSH activity was used as an indicator of apoptosis in the tumor tissue, as GSH depletion is a hallmark of cell death in response to apoptotic stimuli. As shown in Figure 5C, no significant changes in GSH levels were observed in healthy brain tissue exposed to no treatment, blank STH, and Dox-loaded STH, suggesting that the STH itself and the Dox released from Dox-loaded STH stays concentrated in the cancerous region where the STH was injected. Systemic effects (Figure 5D) of Dox are evident in a slightly reduced level of GSH in the heart, which is a common side effect of Dox treatment, and systemic effects of both the blank STH and Dox-loaded STH are observed with reduced GSH levels in the kidneys [31]. However, evidence of systemic impacts from blank STH and Dox-loaded STH suggests that the systemic impacts are minor.

Cytochrome c is a marker of cell death and cell respiration and is often upregulated in cancerous tissue by its role in oxidative phosphorylation and during apoptosis [32]. As shown in Figure 5E, increased cytochrome c levels are observed in cancerous tissues and a minor increase in cytochrome c for blank STH and a large increase for Dox-loaded STH when compared to untreated tumor tissue. The increased levels for treated conditions indicate an increase in apoptosis that would be associated with vascular occlusion for the blank STH and exposure to doxorubicin for the Dox-loaded STH. Systemically, no major changes in cytochrome c activity were observed when comparing untreated mice with treated conditions in evaluated organs (Figure 5F). However, a slight decrease in activity was observed in the liver, which can be attributed to Dox-related liver injury inducing mitophagy as a protective mechanism [33].

Malondialdehyde (MDA) is an indicator of lipid peroxidation from oxidative stress that can be caused by both cancer and treatment with doxorubicin [34,35]. Individual and combinatorial effects from tumor tissue, vascular occlusion, and exposure to doxorubicin account for the slight MDA increase in healthy tissue exposed to Dox-loaded STH compared to the other healthy tissues (Figure 5G) and the increasing levels of MDA for untreated, blank STH, and Dox-loaded STH-treated tumor tissue. Systemic analysis (Figure 5H) indicated no major changes in MDA levels in the organs analyzed.

In summary, the local and systemic mitochondrial dysfunction analysis shows that the major toxic effects of doxorubicin and vascular occlusion by the STH are limited to the tumor tissue region in the brain, and minimal systemic effects that can be attributed to doxorubicin and STH degradation are observed.

#### 3.5.4. Histopathological Evaluation of Dox-Loaded STH Tissue Distribution

To further evaluate the Dox distribution of localized Dox-loaded STH treatment on the other organs of the tumor mice models, histological imaging of the tumor tissue and liver and heart tissues was prepared on day 30 after treatment. According to the H&E-stained images shown in Supplementary Figure S6, the tumor tissue exhibits a cell nuclei lysis footprint, characterized by the smaller cell body and deformation of the cell membrane integrity, after treatment with free Dox and Dox-loaded STH. These findings were not observed in tissue obtained from mice receiving no treatment or treated with the blank gel. Additionally, the liver tissue is almost intact in the localized treatment group, with no cell morphological changes observed in all treatment groups. In evaluating heart tissue, we did find morphological changes in the cell membrane in the presence of the free drug and localized Dox injections. This is likely due to the well-known cardiotoxicity of Dox [36]. Nevertheless, we didn’t observe any significant morphological changes in cardiomyocytes between the free Dox and Dox-loaded STH conditions.

## 4. Conclusions

The present study demonstrates a Laponite, gelatin, and doxorubicin nanocomposite STH as an effective treatment for glioblastoma multiforme and breast cancer. Controlling electrostatic interactions between Dox and the nanocomposite characteristics such as pore size, swelling, and degradation by varying the ratio of gelatin and Laponite allows for effective control of the release kinetics of Dox from the STH. The rheological properties of STH make it a feasible biomaterial that can be used as a local treatment for glioblastoma multiforme or breast cancer, and it has potential for long-term treatment. The antitumor capability of Dox-loaded STHs is shown through multiple drug screening methods, including a biomimicking human glioblastoma-on-a-chip antitumor activity study. Finally, the drug-loaded STH was shown to increase overall survival in rodents inoculated with glioblastoma and breast cancer, proving that this material can be used as an effective method of localized drug delivery for oncologic disease. Future directions for this research include long-term in vivo studies to assess for any potential long-term cytotoxic effects and further optimization of the material formulation and dosage.

## Data Availability

Data can be made available by way of request to the corresponding author.

## Supplementary Materials

The following supporting information can be downloaded at: https://www.mdpi.com/article/10.3390/pharmaceutics15092255/s1, Figure S1: Physical Properties of the drug-eluting nanocomposite hydrogel. (A) SEM images of nanocomposite gel at different nanosilicate to total gel percentage with no drug. (B) SEM images of nanocomposite gel (9NC50) loaded with different concentration of Dox. (C) Degradation profile of the nanocomposite gel compositions in the presence of collagenase. (D) Swelling of the nanocomposite gel compositions in PBS. Error bars are the standard deviation of at least three replicates; Figure S2: Dox-loaded STHs effectively kill MCF7 cells with similar efficacy to free Dox. (A) Free Dox treatment over 96 hours. (B) Treatment with Dox-loaded STHs over 96 hours. (C) 10X Magnification images of cell morphology and infiltration of Dox into cells 48 hours after treatment; Figure S3: Dox-loaded STHs effectively kill U87 cells with similar efficacy to free Dox. (A) Free Dox treatment over 96 hours. (B) Treatment with Dox-loaded STHs over 96 hours. (C) 10X Magnification images of cell morphology and infiltration of Dox into cells 48 hours after treatment; Figure S4: Dox-Loaded STHs show effective DOX penetration of 3D tumor tissue and improved anti-tumor activity. (A) Free Dox treatment of 3D tumor tissue is less effective than in 2D study. (B) Dox-loaded STHs effectively treat 3D tumor tissue and show dependence on the amount of loaded Dox. (C) An untreated, live-dead stained 3D tumor tissue has spindle-shaped cell morphology and high cell density. (D) Live-dead stained cross sections of unloaded and Dox-loaded STH treated tissues show changes in cell morphology and viability with varying treatment; Figure S5: Numerical analysis of drug diffusion in the cancer-on-chip model confirms data observed from experiments. (A) Schematic of Brain-tumor-on-a-chip. (B) Comparison of COMSOL free Dox and 9NC25-150 Dox diffusion simulation with experimental results. (C) Free Dox diffusion normalized intensity signal levels out after 100 minutes and (D) 9NC25-150 Dox diffusion still shows a gradient after 96 hours. (E) Dox normalized intensity over the center channel cross section moments after injection of free Dox solution (left) or Dox-loaded STH (right); Figure S6: (A) H&E staining and (B) relative cell accumulation results extracted from the image analysis of stained tissue sections (n = 15) at the day 30 after the first treatment. (Scale bars = 200 μm).
