# Supplementary material for: A Drug-Eluting Injectable NanoGel for Localized Delivery of Anticancer Drugs to Solid Tumors
Source: Pharmaceutics. 2023 Aug 31;15(9):2255. doi: 10.3390/pharmaceutics15092255 (PMC10534730; doi:10.3390/pharmaceutics15092255)
Supplement: Supplementary file 1 [file pharmaceutics-15-02255-s001.zip › pharmaceutics-2527847-supplementary.pdf]

pharmaceutics

MDPI

Supplementary Materials: Drug-eluting Injectable NanoGel for Localized Delivery of Anticancer Drugs to Solid Tumors

Brent Godau[†,1,2], Sadaf Samimi[†,1,2], Amir Seyfoori[†,1,2], Ehsan Samiei[1,2], Tahereh Khani[3], Parvaneh Naserzadeh, Ali-reza Hassani Najafabadi[5], Emal Lasha[6], Keivan Majidzadeh[7], Behnaz Ashtari[8], Gabriel Charest[9], Christophe Morin[9], David Fortin[9] and Mohsen Akbari[1,2,4,*]

Supplementary information

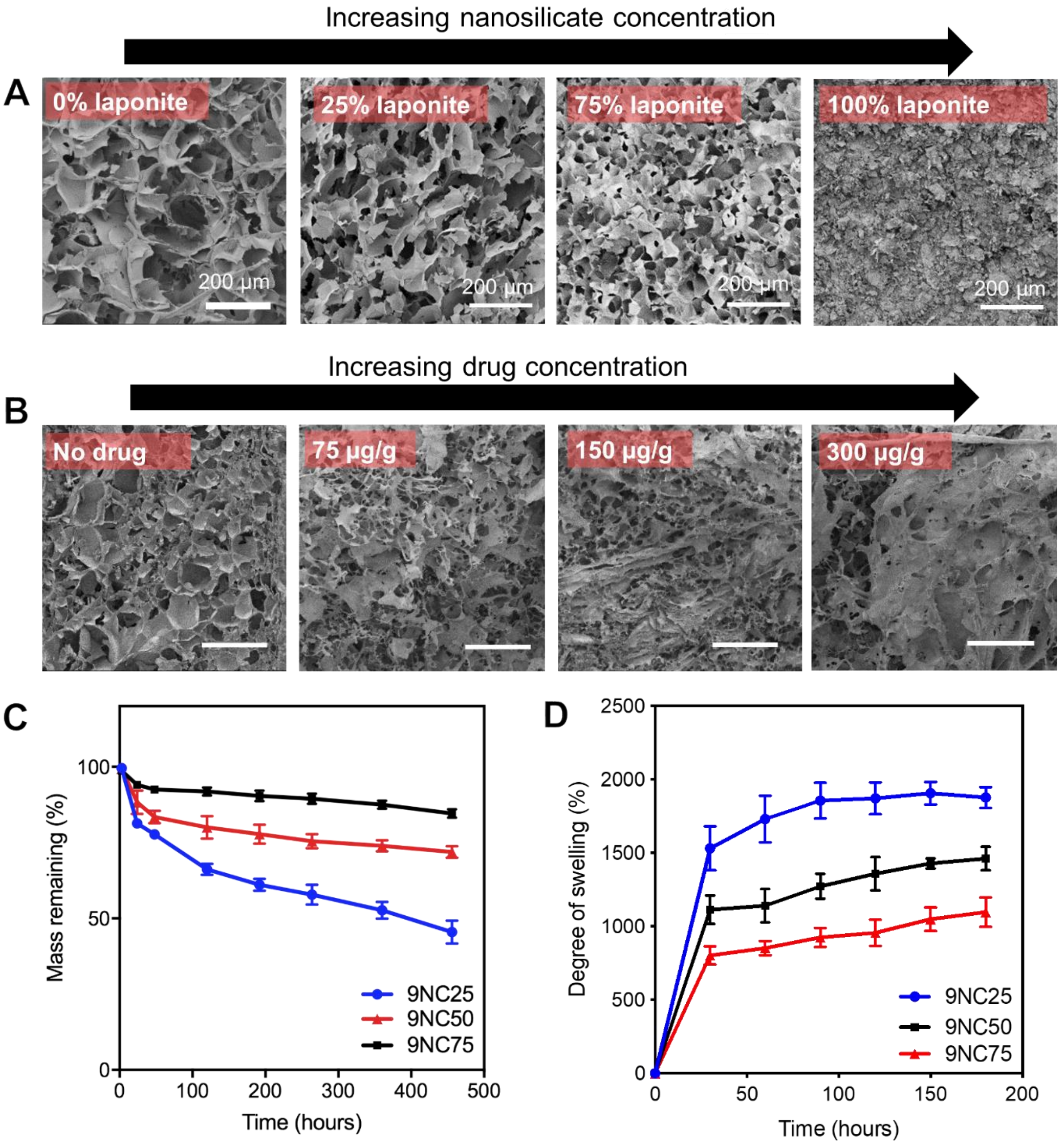

Supplementary Figure S1. Physical Properties of the drug-eluting nanocomposite hydrogel. (A) SEM images of nanocomposite gel at different nanosilicate to total gel percentage with no drug. (B) SEM images of nanocomposite gel (9NC50) loaded with different concentration of Dox. (C) Degradation profile of the nanocomposite gel compositions in the presence of collagenase. (D) Swelling of the nanocomposite gel compositions in PBS. Error bars are the standard deviation of at least three replicates.

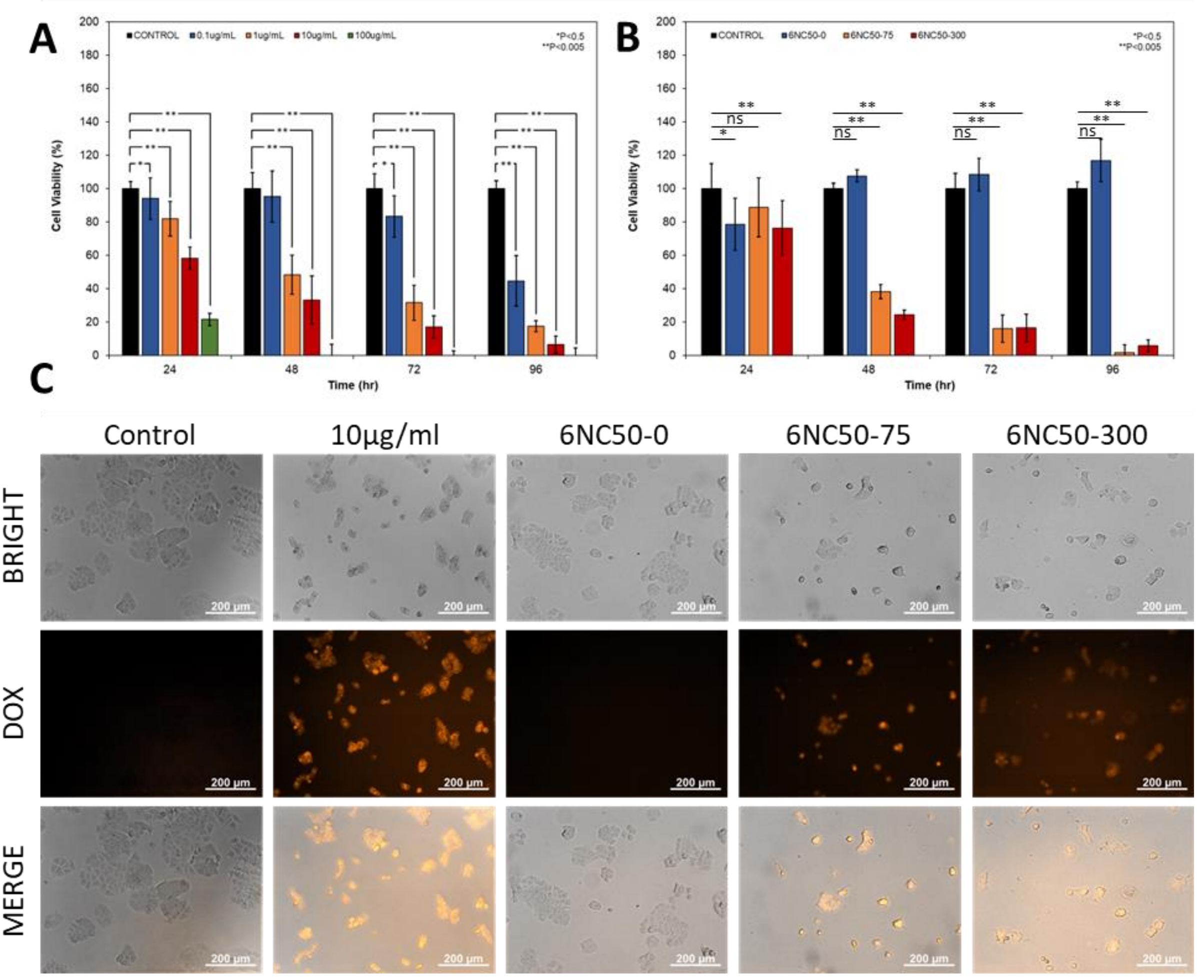

Supplementary Figure S2. Dox-loaded STHs effectively kill MCF7 cells with similar efficacy to free Dox. (A) Free Dox treatment over 96 hours. (B) Treatment with Dox-loaded STHs over 96 hours. (C) 10X Magnification images of cell morphology and infiltration of Dox into cells 48 hours after treatment.

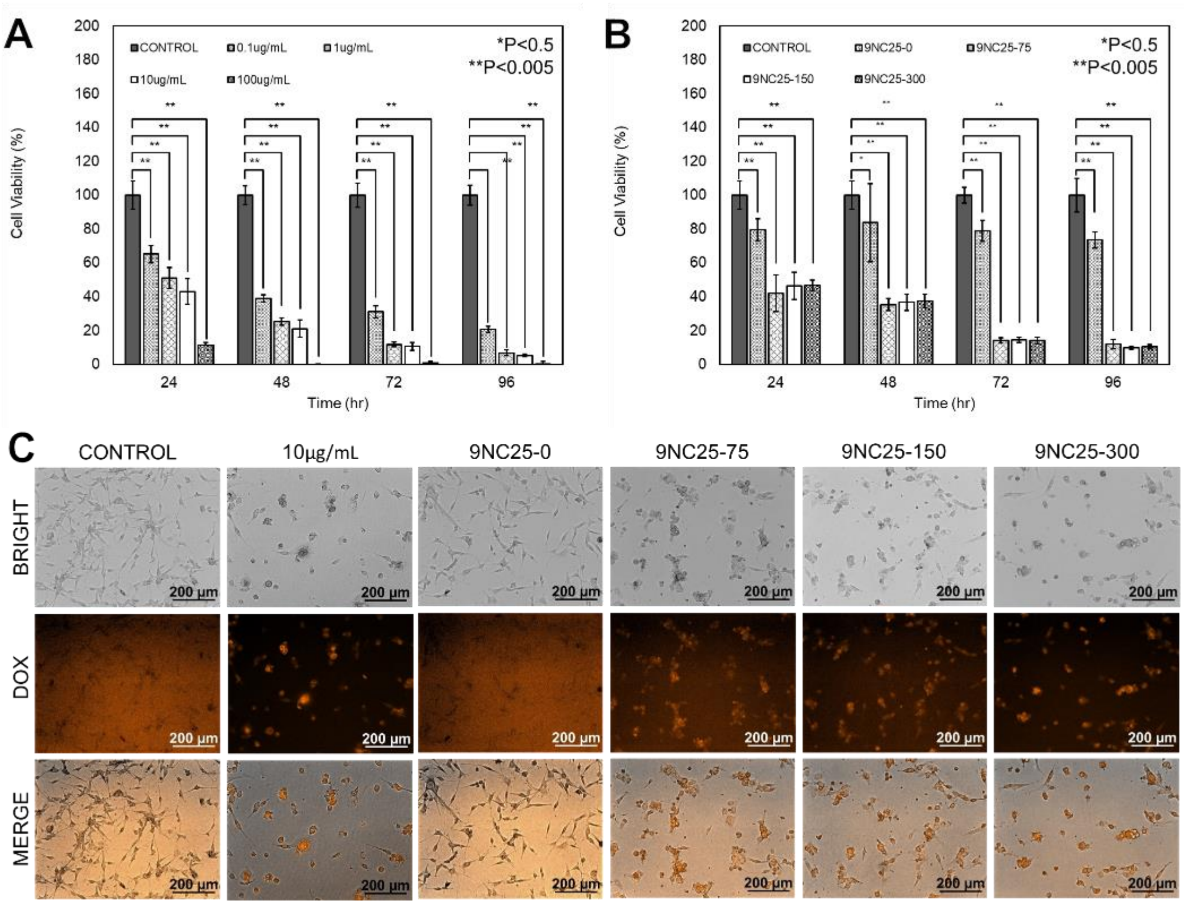

Supplementary Figure S3. Dox-loaded STHs effectively kill U87 cells with similar efficacy to free Dox. (A) Free Dox treatment over 96 hours. (B) Treatment with Dox-loaded STHs over 96 hours. (C) 10X Magnification images of cell morphology and infiltration of Dox into cells 48 hours after treatment.

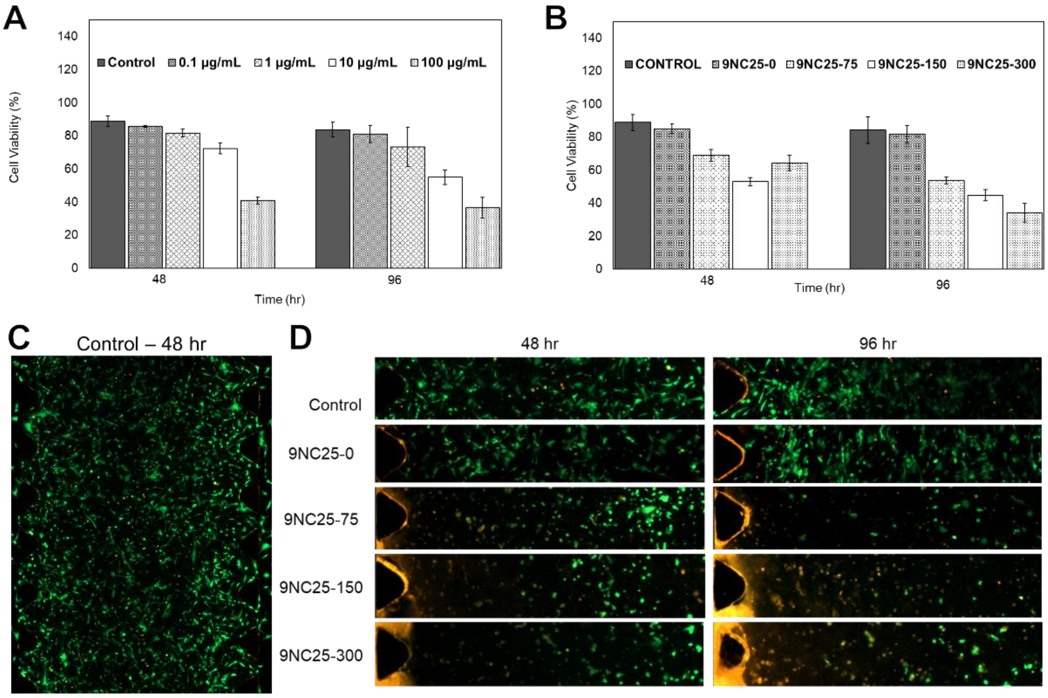

Supplementary Figure S4. Dox-Loaded STHs show effective DOX penetration of 3D tumor tissue and improved anti-tumor activity. (A) Free Dox treatment of 3D tumor tissue is less effective than in 2D study. (B) Dox-loaded STHs effectively treat 3D tumor tissue and show dependence on the amount of loaded Dox. (C) An untreated, live-dead stained 3D tumor tissue has spindle-shaped cell morphology and high cell density. (D) Live-dead stained cross sections of unloaded and Dox-loaded STH treated tissues show changes in cell morphology and viability with varying treatment.

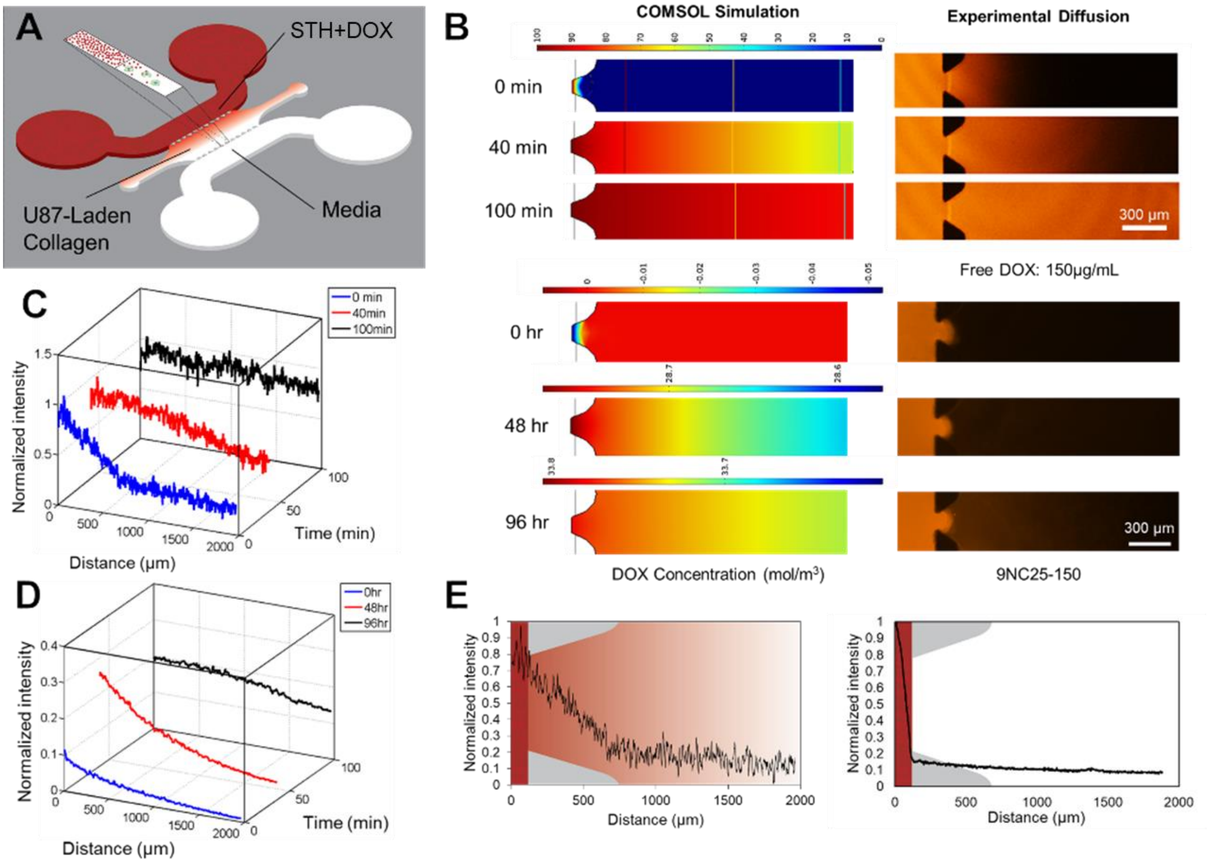

Supplementary Figure S5. Numerical analysis of drug diffusion in the cancer-on-chip model confirms data observed from experiments. (A) Schematic of Brain-tumor-on-a-chip. (B) Comparison of COMSOL free Dox and 9NC25-150 Dox diffusion simulation with experimental results. (C) Free Dox diffusion normalized intensity signal levels out after 100 minutes and (D) 9NC25-150 Dox diffusion still shows a gradient after 96 hours. (E) Dox normalized intensity over the center channel cross section moments after injection of free Dox solution (left) or Dox-loaded STH (right).

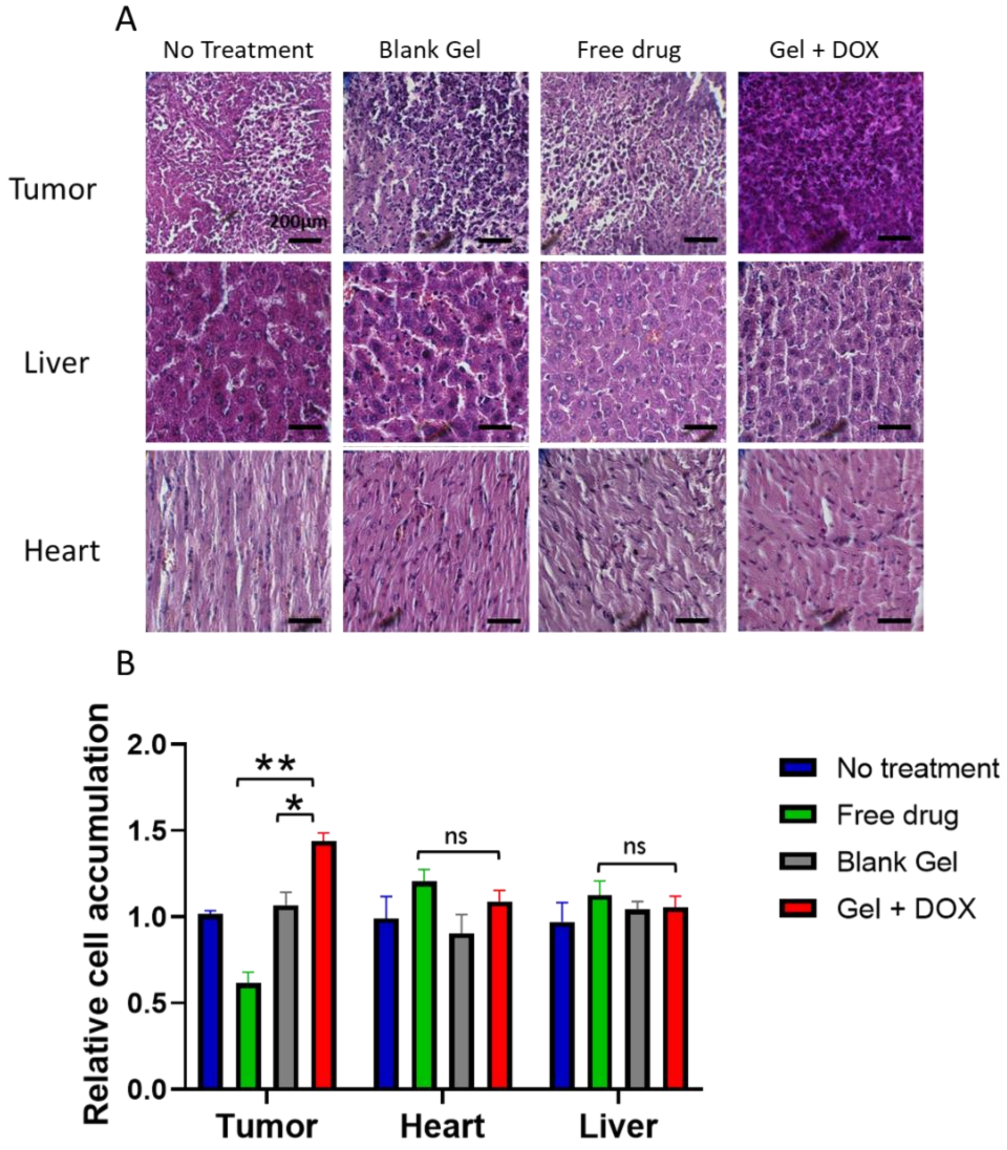

Supplementary Figure S6. A) H&E staining and B) relative cell accumulation results extracted from the image analysis of stained tissue sections (n=15) at the day 30 after the first treatment. (Scale bars = 200 μm).
